# A comparison of growth and dietary adequacy of children with celiac disease on a gluten-free diet with their healthy-peers at a tertiary care center in Turkey

**DOI:** 10.3389/fped.2025.1592342

**Published:** 2025-06-13

**Authors:** Neslihan Ekşi, Rukiye Bozbulut, Eda Köksal, Buket Dalgıç

**Affiliations:** ^1^ Department of Pediatric Gastroenterology, Gazi University Faculty of Medicine, Ankara, Türkiye; ^2^ Department of Pediatric Endocrinology, Gazi University Faculty of Medicine, Ankara, Türkiye; ^3^ Department of Nutrition and Dietetics, Faculty of Health Sciences, Gazi University, Ankara, Türkiye

**Keywords:** celiac disease, gluten-free diet, dietary assessment, nutrient intake, anthropometry

## Abstract

**Introduction:**

Celiac disease (CD) is a chronic autoimmune disorder that requires strict adherence to a gluten-free diet (GFD) initiated after diagnosis. This limited diet may lead to nutritional deficiencies. The aim of this study was to evaluate nutritional intake and dietary adequacy of children with CD having good adherence to a GFD compared with their healthy peers and to assess the contribution of commercial gluten-free products on the daily energy and macronutrient intakes.

**Methods:**

This cross-sectional case-control study included children with CD (age range, 2–18 years) and age- and sex-matched healthy controls. Demographic characteristics, anthropometric measurements and food consumption (3-day food record) were recorded. The groups were compared for dietary compositions, dietary adequacy, and anthropometric parameters.

**Results:**

The study compared 51 patients with 54 controls. The patients had significantly lower height-for-age Z-scores and body mass index-for-age Z-scores (*p* < 0.05). The dietary daily energy, protein, fat and fiber intakes were significantly lower in the patients than in the healthy controls (*p* < 0.05). The mean nutrient adequacy ratio (NAR) for protein, thiamine, calcium, magnesium, iron, zinc and fiber was significantly lower in the patients for both sexes (*p* < 0.05 for all) and the mean NAR for vitamin A and folate was lower in the patients in females (*p* < 0.05 for all). The mean nutrient adequacy ratio (MAR) of protein, thiamine, calcium, magnesium, iron, zinc and fiber was lower in the patients than in the controls (*p* < 0.05 for all).

**Conclusion:**

A comprehensive dietary assessment for patients with CD may enhance their adaptation to healthy nutrition and facilitate their optimal growth.

## Introduction

Celiac disease (CD) is an immune-mediated enteropathy triggered by gluten ingestion in genetically predisposed individuals (1, 2). The prevalence of celiac disease is estimated at approximately 1% in the general population (3). Damage to the mucosa of the small intestine causes malabsorption resulting in nutritional deficiencies (4). A gluten-free diet (GFD) is the sole treatment available for CD (5). The GFD excludes wheat, barley, rye, and their derivatives, including starch, flour, bread, and pasta etc. Adherence to a GFD enables healing of small bowel mucosal damage and restores normal absorption of nutrients within 6 months to 1 year (6). This facilitates the amelioration of clinical symptoms, normalization of laboratory and histological findings, and improvement of disease prognosis (7).

The nutritional adequacy of a GFD after the diagnosis of CD and its effects on the anthropometric parameters of patients with CD have become a compelling issue (8, 9). While some studies have reported positive effects of GFD such as facilitating loss of body fat, attaining a fat-free body mass, providing underweight and overweight patients and accelerating linear growth, other studies have reported negative effects on the body composition and anthropometric parameters of children with CD such as weight gain and obesity (8, 10–13). Elimination of gluten for the production of commercially available gluten-free products (GFPs) leads to alterations in the macro- and micronutrient compositions of foods (7). GFPs tend to contain higher amounts of carbohydrates, fats to improve their palatability and lower amounts of protein, fiber, folate, iron, and vitamin B (thiamine, riboflavin, and niacin) (14, 15). Thus, despite being associated with better outcomes, a GFD may cause unbalanced distributions of carbohydrates, fats, and proteins and inadequate intake of micronutrients (16).

It is crucial to prioritize early adaptation to a strict GFD to enhance the overall health of individuals with CD and promote their healthy growth and development. Hence,the purpose of the present study was threefold: (1) to assess the nutritional intake—including both macro- and micronutrients—and identify possible deficiencies in children with CD who demonstrate good adherence to a GFD, in comparison with healthy peers; (2) to examine gender differences in dietary adequacy and nutritional intake; and (3) to evaluate the impact of a GFD on growth parameters and determine the contribution of commercial gluten-free products (GFPs) to total daily energy and macronutrient intake.

## Materials and methods

### Study design and population

This cross-sectional, case-control study was conducted on children with CD and age- and sex-matched healthy controls in a tertiary hospital in Turkey. Patients were recruited from January 2016 to November 2018 in the Paediatric Gastroenterology Outpatient Clinic. Children aged between 2 and 18 years with a confirmed diagnosis of CD, based on the criteria of the European Society for Paediatric Gastroenterology, Hepatology, and Nutrition (ESPGHAN) were included (17). Only patients who had been on a GFD for at least one year and showed good dietary adherence, confirmed by negative tissue transglutaminase antibody (tTg-IgA) levels and regular follow-up with a dietitian, were enrolled. Exclusion criteria were the presence of a chronic illness such as type 1 diabetes mellitus, hypothyroidism, and IgA deficiency that accompanies CD, receiving enteral or parenteral nutrition, or having any diet restriction other than a GFD. An age- and sex-matched healthy control group was formed from children who were examined for dyspepsia and admitted to the Outpatient Clinic of Pediatrics. Inclusion criteria for the control group were not having a chronic illness, not receiving enteral or parenteral nutrition, and not having special diet restrictions.

### Assessment of dietary adherence

Tissue transglutaminase IgA (tTg-IgA) antibodies were detected using enzyme- linked immunosorbent assay (ELISA) according to the instructions of the manufacturers (Euroimmun, Zedira, Organtech, Germany). For tTg-IgA, the results were considered as normal for <7, an equivocal range for 7–10 and positive for >10 U/ml.

Tissue transglutaminase IgA antibody level was taken into account in the evaluation of dietary compliance; patients with normal tTg-IgA levels (<10 U/ml) were considered as “good adherence to diet” (17). Patients who stated that they consumed gluten-containing products with variable frequency (occasionally) and/or whose tTg-IgA levels were found to be above normal (≥10 U/ml) were excluded from the study.

### Dietary assessment

The participants' dietary assessment was based on a 3-day food record, consisting of two weekdays and one weekend day, completed retrospectively. The dietician explained the children and their families how to fill in the food consumption record. A “Food and Meal Photo Catalog: Measurements and Quantities” was used to determine the quantities and sizes of consumed food and beverages (18). The amounts of the meals' nutrients per portion consumed by each participant were calculated with the help of the “Standardized Food Recipes” book (19). The dietary energy and nutrients were assessed using the “Nutrition Information Systems Package Program” (BeBiS, Ebispro for Windows, Germany; Turkish Version/BebiS 8.2; 2019, İstanbul), a software database that contains food composition tables for all foods (20). BebiS Program is a particular program that has been used for Nutrition and Dietetics for 22 years in Turkey. The program was developed according to the professional standards of academicians and dieticians, ensuring its reliability and validity. It has thousands of food databases based on references to the World Health Organization, Dietary Guidelines for Turkey, and other scientific sources.

### Nutrient adequacy analysis

The recommended daily allowance (RDA) values according to age and sex were used for the assessment of energy and nutrient intake. The nutrient adequacy ratio (NAR) was calculated for 11 nutrients (protein, vitamins A, E and C, thiamine, folate, calcium, magnesium, iron, zinc, and fiber) using the dietary reference intake (DRI) recommended percentages with the following formula: NAR (%) = (nutrient intake of an individual/DRI of the nutrient) × 100 (21). The mean adequacy ratio (MAR, %) was calculated by dividing the sum of each NAR by the number of nutrients with the following formula: MAR (%) = sum of NAR (%) for each nutrient/number of nutrients. For both NAR and MAR a value of 100% is the ideal since it means that the intake is the same as the requirement (22). Daily energy and macronutrient intakes from GFPs in patients with CD were also evaluated using the information on the labels of the GFPs.

### Anthropometric measurements

Body weight and height measurements of the patients and controls were performed by the same dietitian. Body weight was measured with the subjects wearing minimal clothing to the nearest 0.1 kg using a digital scale. Length measurement was performed with the subjects without shoes to the nearest 0.1 cm using a stadiometer. The BMI was calculated as body weight (in kg) divided by the square of height (in m^2^) and expressed as kg/m^2^. Height-for-age Z-score (HAZ), weight-for-age Z-score (WAZ), and BMI-for-age Z-score (BAZ) were calculated according to the standards defined by the World Health Organization (WHO) (23).

### Statistical analysis

Data analysis was performed using the IBM SPSS Statistics for Windows, version 21 (IBM Corp., Armonk, NY, USA). Descriptive statistics were used to report frequencies, percentages, and either means ± standard deviation (SD) or medians (minimum and maximum) for continuous variables, depending on data normality as assessed by the Kolmogorov–Smirnov test. According to the normality test, age, anthropometric measurements and indices, along with energy and MAR values showed normal distribution. Normally distributed variables were analyzed using the independent samples *t*-test, while non-normally distributed variables were analyzed using the Mann–Whitney *U*-test, where appropriate. A *P* value of <0.05 was considered to be statistically significant.

### Ethical approval

The study was approved by the Clinical Research Ethics Committee of Gazi University, Ankara, Turkey (approval number: 25901600-598; date: 14.12.2015). Informed consent was obtained from the legal guardians of all participating children, and assent was obtained from each subject.

## Results

### Demographic and clinical characteristics

The study included 51 children with CD and 54 healthy controls. The mean age of 51 celiac patients (34 female, 66.7%) and 54 controls (28 female, 51.9%) was 10.2 ± 3.79 years and 11.0 ± 3.81 years, respectively. The patients and controls did not significantly differ regarding age (*p* = 0.89) and sex (*p* = 0.123). The median age at diagnosis was 8 (1–12.5) years for males and 4.7 (1.5–14) years for females in the patient group. The median duration of GFD was 31.8 (15–83) months in the patient group.

### Anthropometric measurements

The mean body weight, BMI, HAZ, and BAZ values significantly differed between the patients and controls (*p* < 0.05 for all; Table 1); the children with CD had significantly lower HAZ and BAZ values (Table 1). The mean body weight, BMI, WAZ, HAZ and BAZ values of the males with CD and the mean BAZ value of the females with CD were significantly lower as compared with their healthy control group (Table 1).

**Table 1:** Age and anthropometric measurements in children with celiac disease and healthy controls according to sex.

Variable	Study groups, mean ± SD			Male, mean ± SD			Female, mean ± SD		
	Celiac patients *n* = 51	Control group *n* = 54	*P*	Celiac patients *n* = 17	Control group *n* = 26	*P*	Celiac patients *n* = 34	Control group *n* = 28	*P*
Age, years	10.2 ± 3.79	11.0 ± 3.81	0.89	11.6 ± 3.30	11.2 ± 3.11	0.62	10.5 ± 4.23	10.8 ± 4.41	0.76
Height, cm	135.9 ± 18.94	141.9 ± 21.64	0.13	140.6 ± 17.71	144.9 ± 19.4	0.46	133.6 ± 19.36	139.2 ± 23.52	0.31
Body weight, kg	33.2 ± 13.21	41.9 ± 18.27	**0** **.** **006**	34.3 ± 12.6	44.4 ± 17.87	0.048	32.7 ± 13.85	39.6 ± 18.65	0.109
BMI, kg/m^2^	17.1 ± 3.26	19.5 ± 3.47	**<0** **.** **001**	16.7 ± 2.88	20.1 ± 3.32	0.002	17.3 ± 3.45	19.0 ± 3.58	0.067
WAZ	−0.33 ± 1.31	0.19 ± 0.91	0.126	−1.05 ± 0.29	0.42 ± 0.91	0.008	−0.15 ± 1.41	−0.04 ± 0.88	0.805
HAZ	−0.89 ± 1.48	−0.15 ± 0.91	**0** **.** **002**	−1.02 ± 1.31	−0.09 ± 0.87	0.008	−0.82 ± 1.57	−0.20 ± 0.97	0.072
BAZ	−0.50 ± 1.38	0.65 ± 0.86	**<0** **.** **001**	−0.78 ± 1.28	0.98 ± 0.87	<0.001	−0.36 ± 1.42	0.34 ± 0.74	**0** **.** **021**

BMI, body mass index; WAZ, weight-for-age Z-score; HAZ, height-for-age Z-score; BAZ, body mass index-for-age Z-score.

Values with *P* < 0.05 are shown in bold.

### Energy and macronutrient intake

The daily energy and macronutrient intakes in the patients and controls according to sex are presented in Table 2. Accordingly, the mean daily dietary energy, protein, fat and fiber intakes and the percentage of energy obtained from protein were lower in the patients than in the controls for both sexes (*p* < 0.05 for all). The mean daily carbohydrate intakes were lower (only significant for females) but the percentage of energy obtained from carbohydrates was higher (although not significant) in patients with CD than in the controls for both sexes. The mean daily fat intake was significantly lower in the patients than in the controls for both males (*p* = 0.006) and females (*p* = 0.001); however, the percentage of energy obtained from fats was similar in the patients and controls for both sexes. The percentages of total energy obtained from saturated fatty acids, monounsaturated fatty acids (MUFAs), and polyunsaturated fatty acids (PUFAs) did not significantly differ between the patients and controls for both sexes (*p* > 0.05 for all). The mean daily cholesterol intake was significantly lower in patients with CD than in the controls for both sexes (*p* < 0.05 for all).

**Table 2:** Daily energy and macronutrient intakes in children with celiac disease and healthy controls according to sex.

	Male, Mean ± SD			Female, Mean ± SD		
	Celiac patients *n* = 17	Control group *n* = 26	*P*	Celiac patients *n* = 34	Control group *n* = 28	*P*
Total energy, kcal	1,475.9 ± 524.61	1,889.7 ± 461.92	**0** **.** **009**	1,356.9 ± 298.98	1,669.9 ± 392.87	**<0** **.** **001**
Macronutrients						
Carbohydrate, g	179.3 ± 72.65	208.8 ± 66.96	0.179	161.6 ± 49.67	183.1 ± 51.28	**0** **.** **023**
E% carbohydrate	48.8 ± 7.43	45.7 ± 5.80	0.134	47.4 ± 10.11	44.8 ± 5.74	0.318
Protein, g	49.0 ± 18.68	77.0 ± 26.28	**<0** **.** **001**	43.1 ± 10.71	67.7 ± 17.51	**<0** **.** **001**
Protein, g/kg	1.53 ± 0.62	1.84 ± 0.48	0.074	1.61 ± 0.86	1.99 ± 0.76	0.067
E% Protein	13.7 ± 3.09	16.5 ± 2.93	**0** **.** **005**	12.7 ± 3.04	16.6 ± 2.18	**<0** **.** **001**
E% fat	37.6 ± 6.16	37.8 ± 5.64	0.905	37.5 ± 7.74	38.7 ± 5.51	0.667
Saturated fatty acids, g	21.7 ± 8.76	27.5 ± 9.08	**0** **.** **042**	19.7 ± 6.78	25.7 ± 9.17	**0** **.** **002**
E% saturated fatty acids	13.2 ± 2.15	13.3 ± 3.43	0.943	13.1 ± 3.79	13.7 ± 3.22	0.818
MUFAs, g	20.7 ± 7.14	27.1 ± 8.82	**0** **.** **015**	19.98 ± 5.09	25.5 ± 7.95	**<0** **.** **001**
E% MUFAs	12.8 ± 2.28	13.1 ± 3.47	0.402	13.4 ± 2.80	13.7 ± 2.90	0.586
PUFAs, g	13.5 ± 5.53	16.6 ± 5.69	0.081	13.9 ± 5.25	15.8 ± 4.72	0.110
E% PUFAs	8.7 ± 3.23	7.9 ± 2.06	0.682	9.3 ± 2.68	8.6 ± 2.17	0.133
Cholesterol, mg	234.9 ± 91.50	315.6 ± 131.25	**0** **.** **033**	205.3 ± 103.09	293.1 ± 84.51	**0** **.** **001**
Fiber, g/1,000 kcal	12.8 ± 5.52	17.7 ± 6.74	**0** **.** **017**	10.9 ± 3.36	17.8 ± 6.09	**<0** **.** **001**

%E, percentage of total energy; MUFAs, monounsaturated fatty acids; PUFAs, polyunsaturated fatty acids.

Values with *P* < 0.05 are shown in bold.

### Micronutrient intake

The median daily micronutrient intakes in patients and controls according to sex are presented in Table 3. Accordingly, the median daily intakes of thiamine and riboflavin were significantly lower in patients with CD compared to controls for both sexes (*p* < 0.05 for all). Among females, the median daily intakes of vitamins A, B6 and C, niacin and folate were also significantly lower in the patient group than in the controls (*p* < 0.05 for all). Furthermore, both male and female patients exhibited significantly lower median intakes of calcium, magnesium, phosphorus, and selenium (*p* < 0.05 for all), whereas potassium, iron, and zinc intakes were significantly lower only among female patients (*p* < 0.05 for all).

**Table 3:** Daily micronutrient intakes in children with celiac disease and healthy controls according to sex.

Micronutrients	Male, median (IQR)			Female, median (IQR)		
	Celiac patients *n* = 17	Control group *n* = 26	*P*	Celiac patients *n* = 34	Control group *n* = 28	*P*
itamin A, µg	654.8 (957.6)	739.7 (593.7)	0.587	497.1 (339.90)	847.3 (455.9)	**<0** **.** **001**
Thiamine, mg	0.58 (0.31)	0.83 (0.34)	**0** **.** **002**	0.56 (0.19)	0.86 (0.21)	**<0** **.** **001**
Riboflavin, mg	1.13 (0.60)	1.4 (0.73)	**0** **.** **005**	0.96 (0.43)	1.39 (0.70)	**<0** **.** **001**
Niacin, mg	9.5 (13.82)	12.8 (12.11)	0.419	8.2 (6.75)	12.9 (5.23)	**0** **.** **003**
Vitamin E, mg	13.3 (5.28)	15.2 (7.85)	0.853	15.5 (7.23)	13.2 (8.67)	0.115
Vitamin B6, mg	0.90 (0.71)	1.2 (0.55)	0.198	0.84 (0.41)	1.12 (0.34)	**0** **.** **002**
Folate, µg	209.0 (99.58)	242.2 (165.98)	0.305	187.2 (75.48)	247.6 (86.30)	**<0** **.** **001**
Vitamin B12, µg	3.5 (4.47)	4.9 (3.36)	0.198	3.6 (2.5)	4.97 (3.22)	0.441
Vitamin C, mg	63.7 (76.20)	76.4 (59.83)	0.786	59.7 (37.49)	82.9 (63.88)	**0** **.** **002**
Calcium, mg	528.5 (242.34)	717.2 (408.10)	**0** **.** **001**	501.1 (183.0)	730.7 (413.57)	**<0** **.** **001**
Potassium, mg	1,821.9 (1,312.90)	2,349.0 (836.8)	0.060	1,670.8 (620.8)	2,331.7 (591.2)	**<0** **.** **001**
Magnesium, mg	172.1 (87.10)	259.9 (84.2)	**0** **.** **001**	161.4 (41.2)	242.3 (74.6)	**<0** **.** **001**
Phosphorus, mg	859.6 (400.80)	1,124.5 (491.44)	**0** **.** **002**	753.4 (248.48)	1,114.9 (378.78)	**<0** **.** **001**
Iron, mg	6.5 (4.51)	9.2 (2.88)	0.069	7.1 (2.66)	9.2 (4.04)	**<0** **.** **001**
Zinc, mg	7.3 (5.70)	8.8 (4.21)	0.078	7.1 (2.71)	8.9 (4.0)	**<0** **.** **001**
Selenium, µg	1.46 (3.75)	4.8 (5.75)	**0** **.** **024**	0.73 (3.95)	3.4 (6.44)	**0** **.** **006**

IQR, The interquartile range.

Values with P < 0.05 are shown in bold.

### Nutrient adequacy ratio (NAR) and mean adequacy ratio (MAR)

The comparisons of NAR and MAR between the patients and controls according to sex are presented in Table 4. The mean NAR for protein, thiamine, calcium, magnesium, iron, zinc and fiber was significantly lower in the patients for both sexes (*p* < 0.05 for all) and the mean NAR for vitamin A and folate was lower in the patients in females (*p* < 0.05 for all). The MAR of the patients with CD was significantly lower as compared with the controls for both males (*p* = 0.004) and females (*p* < 0.001). Additionally, the MAR values of both patients and controls were lower than the optimal adequacy ratio (a MAR value of 100%) for both sexes.

**Table 4:** Comparisons of the nutrient adequacy ratio (NAR) and mean adequacy ratio (MAR) in patients with celiac disease and healthy controls according to sex.

	Male, mean ± SD			Female, mean ± SD		
	Celiac patients *n* = 17	Control group *n* = 26	*P*	Celiac patients *n* = 34	Control group *n* = 28	*P*
NAR, %						
Energy	94.9 ± 32.72	99.4 ± 14.30	0.535	98.7 ± 27.72	109.8 ± 15.96	0.066
Protein	84.7 ± 18.58	99.4 ± 2.26	**<0** **.** **001**	89.8 ± 12.84	98.9 ± 5.85	**0** **.** **001**
Vitamin A	76.8 ± 23.80	83.1 ± 20.71	0.370	71.9 ± 24.78	94.2 ± 13.94	**<0** **.** **001**
Vitamin E	90.0 ± 14.85	89.9 ± 14.24	0.975	93.7 ± 14.82	86.9 ± 19.27	0.126
Thiamine	52.5 ± 16.86	76.6 ± 15.36	**<0** **.** **001**	59.4 ± 13.98	86.0 ± 14.85	**<0** **.** **001**
Folate	57.4 ± 18.78	66.1 ± 21.43	0.183	53.6 ± 17.85	68.1 ± 15.68	**0** **.** **001**
Vitamin C	81.2 ± 22.36	85.1 ± 23.88	0.601	85.0 ± 22.05	93.8 ± 15.62	0.082
Calcium	39.5 ± 15.38	59.5 ± 19.76	**0.001**	37.6 ± 12.92	59.0 ± 21.47	**<0** **.** **001**
Magnesium	46.0 ± 16.43	68.7 ± 17.21	**<0** **.** **001**	52.1 ± 17.18	77.4 ± 18.68	**<0** **.** **001**
Iron	70.1 ± 20.97	86.5 ± 16.61	**0** **.** **007**	57.7 ± 22.50	76.7 ± 19.62	**0** **.** **001**
Zinc	70.7 ± 23.04	85.1 ± 14.71	**0** **.** **016**	76.9 ± 19.31	89.5 ± 16.10	**0** **.** **008**
Fiber	42.6 ± 17.62	58.9 ± 18.67	**0** **.** **007**	42.5 ± 13.10	67.6 ± 19.33	**<0** **.** **001**
MAR, %	64.7 ± 15.51	78.1 ± 12.77	**0** **.** **004**	65.5 ± 12.39	81.7 ± 10.91	**<0** **.** **001**

NAR, nutrient adequacy ratio; MAR, mean adequacy ratio.

Values with *P* < 0.05 are shown in bold.

### Contribution of gluten-free products (GFPs) to daily intake

Evaluation of daily energy and macronutrient intakes from GFPs in the patients revealed that the GFPs fulfilled an average of 21.45 ± 6.38% of the daily total energy intake of males with CD and an average of 19.53 ± 8.54% daily total energy intake of females with CD (Table 5). The mean percent energy provided by the carbohydrate content of the GFPs was 39.83 ± 8.99% for males with CD and 37.20 ± 14.91% for females with CD.

**Table 5:** Daily total energy and macronutrient intakes from gluten-free products in children with celiac disease according to sex.

Nutrient/Unit	Children with celiac disease, mean ± SD	
	Male (*n* = 17)	Female (*n* = 34)
Energy, kcal	338.57 ± 215.01	266.89 ± 140.34
Energy, %	21.45 ± 6.38	19.53 ± 8.54
Protein, g	2.29 ± 1.94	1.58 ± 0.79
Protein, %	4.56 ± 2.41	3.77 ± 1.90
Fat, g	2.96 ± 3.33	1.81 ± 1.24
Fat, %	4.35 ± 2.89	3.33 ± 2.57
Carbohydrate, g	74.56 ± 44.55	60.13 ± 32.07
Carbohydrate, %	39.83 ± 8.99	37.20 ± 14.91
Fiber, g	2.74 ± 1.58	2.12 ± 1.22
Fiber, %	21.66 ± 7.70	19.94 ± 10.30

## Discussion

This cross-sectional case-control study is important to evaluate dietary intakes, dietary adequacy, and growth parameters in children with CD as compared with their healthy control peers at our hospital. The study demonstrated that the HAZ and BAZ values and energy and macro- and micronutrient intakes of the patients with CD were lower as compared with their healthy peers. Moreover, the MAR values showed that the diet of both groups was lower than optimal dietary adequacy (MAR < 100%) and the dietary adequacy was poorer in the patients.

In the present study, the mean HAZ and BAZ values of patients with CD were lower when compared with those of healthy controls. The mean BAZ values of females with CD and the mean WAZ, HAZ and BAZ values of males with CD were also significantly lower as compared with their healthy peers. Previous studies evaluating anthropometric parameters of patients with CD on a GFD have reported different outcomes (10, 11, 24). Similar to the results of this study, a study from Sweden found that males with CD had lower body weights (lower z-scores and percentiles) and females with CD had lower height percentiles as compared with controls (1). Another study comparing children with CD and healthy controls demonstrated that patients with CD had lower body weights and BMI z-scores despite their high consumption of calories and fat (25). Although our study had similar anthropometric results to the above-mentioned studies, as compared with their healthy peers, the children with CD were observed to have lower intakes of energy, protein, and fat and poorer dietary adequacy, which could be the underlying reason for lower body weight-height percentiles, BMI, and mean HAZ and BAZ values observed in our patients. Different outcomes reported in the literature might be due to differences in the study parameters, such as durations of GFDs during anthropometric evaluations, disease duration, and socioeconomic level. Although having good dietary adherence was one of the inclusion criteria for patients with CD in this study, the patients were unable to achieve a balanced nutrient intake due to GFDs that likely included inappropriate food choices.

The mean daily dietary energy, carbohydrate, protein, fat and fiber intakes were significantly lower in the children with CD than in their healthy control peers. Although not statistically significant, the percentage of energy obtained from carbohydrates was slightly higher in the patients than in the controls; nevertheless, the percentage of energy obtained from carbohydrates was higher than the recommended daily carbohydrate intakes for both groups (21).

The present study revealed that the amount of fat intake was significantly lower in the patients with CD than in the controls for both sexes; however, the percentage of energy obtained from fats was similar for the patients and controls for both sexes and higher than the recommended. Numerous studies have found that the total fat intake of children with CD is significantly higher than healthy controls (1, 25–27). Nowadays, lifestyle changes resulting from economic development and globalization lead children to consume more fast food and unhealthy snacks with high amounts of fat (28). GFPs tend to contain more fat as compared with gluten-containing foods; moreover, carbohydrate restriction in diets of patients with CD may also increase the percentage of energy intake from fat.

The patterns of fatty acid consumption are as important as the total amount of dietary fat (29). In this study, although children with CD had lower intakes of saturated fat, MUFAs and PUFAs for both sexes, no significant difference was found between the patients and controls regarding the percentage of total energy intake from saturated fatty acids, MUFAs and PUFAs for both sexes. The control group had higher saturated fat intake and the amount of saturated fat intakes were above the recommended level both for patients and controls (21). This finding may reflect the imbalance in the intake of fat subtypes in the general population rather than being related to a GFD (30). MUFAs and PUFAs can reduce total cholesterol and LDL cholesterol more than saturated fat (29). In this study, the cholesterol intake of patients with CD was significantly lower than that of the controls for both sexes. While cholesterol intake was below the recommended level in patients with CD, it was above the recommended level for healthy control males. Unlike our findings, a previous study reported that cholesterol intake was above the recommended level for both patients with CD and healthy controls (31).

The daily amount of protein intake, the amount of protein per body weight, and the percentage of energy from protein were lower in the patients than in the controls in this study. It was observed that protein intake of patients with CD was below the DRI levels and protein intake of controls was at the recommended levels. Similar to our study, a number of previous studies have also reported that patients with CD consume less protein as compared with their healthy peers (1, 26, 31, 32). However, there are also other studies reporting no difference in protein intake between patients with CD and healthy controls (24, 25, 33, 34). Varying outcomes obtained from these studies may be due to the differences in the dietary habits of control groups and variability in GFPs in different countries.

In the present study, fiber content of cereals is higher than that of other foods. Several studies have reported fiber intake level of children who are on diets with or without gluten to be below the recommended level (1, 26). Similarly, daily fiber intakes of patients and controls were also found to be below the recommended daily level (25 g/day) in the current study (21). Moreover, the mean daily fiber intake of patients with CD was significantly lower than that of the controls. The reasons underlying this finding may be due to reduced nutritional value of GFPs due to the refining process of flours making up the GFPs, imbalanced dietary habits and low vegetable and fruit consumption in patients with CD.

Grains are among the richest sources of B vitamins; thus, their elimination from a GFD may result in an insufficient intake of B vitamins (35). Our findings demonstrated that thiamine and riboflavin intakes of patients with CD were lower than those of controls for both sexes and that vitamin A, niacin, vitamin B6, folate and vitamin C intakes of females with CD were significantly lower than those of healthy control females. Similarly, another study reported that thiamine, riboflavin, vitamin B6, and niacin intakes were lower in children with CD than in their peers without CD (31). A previous study investigating GFPs by particularly focusing on their thiamine, riboflavin, and niacin contents reported that the GFPs were insufficient in their thiamine, riboflavin, and niacin contents (35). In this study, folate and vitamin C intakes of females with CD were significantly lower than that of healthy control females. While some studies have reported folate intake of patients with CD to be lower than that of the children on a normal diet as was in our study, some other studies have reported folate intake of patients with CD to be at the recommended levels (16, 24, 25, 33).

It has been suggested that patients with CD may experience mineral deficiencies such as iron, calcium, and zinc deficiencies at the time of diagnosis and such as iron, calcium, selenium, zinc, and magnesium deficiencies while on a GFD (36). GFPs tend to contain less calcium, iron, zinc, magnesium, and potassium (37). Some studies have reported that children and adolescents with CD have lower micronutrient intakes (iron, calcium, phosphorus, magnesium, zinc, and selenium) as compared with their healthy peers and with the recommended daily intake levels (1, 27).Within the scope of this study, calcium, magnesium, phosphorus, and selenium intakes of patients with CD were lower for both sexes as compared with their healthy peers and potassium, iron and zinc intakes of females with CD were significantly lower than healthy control females. The reason for the insufficient calcium intake of patients with CD may be due to their consumption of fewer calcium sources than recommended. Additionally, lower protein intake of patients with CD may be the reason for lower intakes of phosphorus in patients with CD.

The current analysis evaluated dietary adequacy of the patients and controls using the NAR (%) and MAR (%) values (22). The MAR value was found significantly lower in patients with CD than in the controls for both sexes. Moreover, the MAR values of both patients and controls were below the optimal dietary adequacy (a MAR value of 100%), indicating that the diets of both patients with CD and healthy controls should be improved. Similarly, a previous study also reported that children and adolescents on a GFD had poor dietary quality (24).

The GFPs in the markets are commonly processed foods. Elimination of storage proteins from foods alters the macro- and micronutrient contents and nutritional values (7). GFPs have generally higher carbohydrate and fat content but lower vitamin B, folate, magnesium, and iron content (31, 37). In our study, evaluation of daily total energy and macronutrient intakes from the GFPs using the information on their labels revealed that GFPs fulfilled an average of 21.5% of the daily total energy intake of males with CD and an average of 19.5% daily total energy intake of females with CD. The mean percentage of energy derived from the carbohydrate content of gluten-free products (GFPs) was found to be relatively high among both male and female children with CD. The GFPs contributed only modestly to the overall dietary fiber intake in children with CD, regardless of sex. This finding is consistent with previous research indicating that many commercially available GFPs are low in fiber due to the refinement of gluten-free flours, the absence of whole grains in their formulation, and their reliance on high-carbohydrate ingredients with low protein and fiber content (36, 38).

To the best of our knowledge, the current study is the first to evaluate detailed nutritional assessment and dietary adequacy among children diagnosed with CD in Turkey. The strengths of this study were the comparison of nutritional status and growth between children with CD and their healthy peers, evaluation of dietary adequacy of both patients and controls by recording their three-day nutrition intake, and evaluation of the contribution of commercial GFPs on the daily energy and macronutrient intakes in patients with CD. On the other hand, one of the limitations of this research is that it was carried out at a single center, which limits the generalizability of the results to the broader population, including children with CD. Another limitation was that we were unable to access the complete vitamin and mineral compositions of certain GFPs in the national nutrient composition database, which resulted in an incomplete evaluation of vitamin and mineral intake in patients with CD. In addition, the necessity of conducting further studies with larger number of patients should be considered.

This study revealed that, despite good adherence to a GFD, children with CD experienced both nutritional inadequacies and excessive intakes. Nutritional assessment of the control group also revealed similar findings. Furthermore, the study demonstrated that the macronutrient composition of GFPs was imbalanced for patients with CD. To address these deficiencies, commercial GFPs should be fortified with fiber, iron, folate, calcium, zinc, B vitamins, and vitamin D. Additionally, the GFD, which is often high in fat and low in fiber, should be modified to ensure that children receive a balanced and adequate diet that supports their normal growth and development.

In conclusion, these results underscore the importance of not only ensuring proper adherence to a lifelong GFD but also addressing potential nutritional imbalances. Therefore, it is crucial to provide comprehensive dietary education and support to children diagnosed with CD and healthy children, with a focus on optimizing their nutrient intake. Furthermore, the study highlights the need for improved formulation of GFPs, particularly by increasing their fiber content, as many current products lack sufficient fiber. In addition, dietary counseling should emphasize the consumption of naturally gluten-free, fiber-rich foods such as fruits, vegetables, legumes, and pseudocereals like quinoa and buckwheat, to help ensure adequate fiber intake and promote better long-term health outcomes for children with CD.

## Data Availability

The raw data supporting the conclusions of this article will be made available by the authors, without undue reservation.
